# Designing and validating an autoverification system of biochemical test results in Hatay Mustafa Kemal University, clinical laboratory

**DOI:** 10.11613/BM.2022.030704

**Published:** 2022-08-05

**Authors:** Bahar Ünlü Gül, Oğuzhan Özcan, Serdar Doğan, Abdullah Arpaci

**Affiliations:** 1 Department of Medical Biochemistry, Kars Harakani Public Hospital, Kars, Turkey; 2 Department of Medical Biochemistry, Hatay Mustafa Kemal University, Hatay, Turkey

**Keywords:** automation, biochemistry, laboratory organization and management, validation/evaluation

## Abstract

**Introduction:**

Autoverification (AV) is a postanalytical tool that uses algorithms to validate test results according to specified criteria. The Clinical and Laboratory Standard Institute (CLSI) document for AV of clinical laboratory test result (AUTO-10A) includes recommendations for laboratories needing guidance on implementation of AV algorithms. The aim was to design and validate the AV algorithm for biochemical tests.

**Materials and methods:**

Criteria were defined according to AUTO-10A. Three different approaches for algorithm were used as result limit checks, which are reference range, reference range **±** total allowable error, and 2nd and 98th percentile values. To validate the algorithm, 720 cases in middleware were tested. For actual cases, 3,188,095 results and 194,520 reports in laboratory information system (LIS) were evaluated using the AV system. Cohen’s kappa (κ) was calculated to determine the degree of agreement between seven independent reviewers and the AV system.

**Results:**

The AV passing rate was found between 77% and 85%. The highest rates of AV were in alanine transaminase (ALT), direct bilirubin (DBIL), and magnesium (Mg), which all had AV rates exceeding 85%. The most common reason for non-validated results was the result limit check (41%). A total of 328 reports evaluated by reviewers were compared to AV system. The statistical analysis resulted in a κ value between 0.39 and 0.63 (P < 0.001) and an agreement rate between 79% and 88%.

**Conclusions:**

Our improved model can help laboratories design, build, and validate AV systems and be used as starting point for different test groups.

## Introduction

Clinical laboratories are centres where millions of tests are analysed that guide the diagnosis and treatment of patients. The workload of clinical laboratories is increasing day by day because of the expansion of test panels, increasing numbers of samples analysed, high-quality expectations, and aiming to report test results in a shorter time. This increased workload causes clinical biochemists to allocate a large part of their working hours for the manual verification of test results (*1*).

The result verification process, which is the most important control step of the postanalytical phase, can be performed in two ways which are manually or by using autoverification (AV) systems. The results are evaluated by the laboratory technician and then by the clinical biochemist (*2*). The appropriate results are verified and sent to the relevant clinic *via* the laboratory information system (LIS). Corrective actions are initiated for the discordant results. These actions are carried out in three phases preanalytical, analytical, and postanalytical according to the type of laboratory errors. Common preanalytical errors include haemolysed sample, clotted sample, inadequate volume, wrong sample tube, and incorrect identification. At the analytical phase, quality control (QC) or calibration failure, interference, and sample mix-ups errors can occur. Some postanalytical errors include delay in reporting, incorrect calculation, and critical results being delayed or not reported. The evaluation of each parameter at laboratory phases can be quite time-consuming for laboratories where the number of experts is insufficient compared to the number of tests that are ordered. Additionally, the evaluation process is subjective because it depends on the personnel and does not include a standardisation. There is a need for AV systems that use multiple algorithms in accordance with the developing technology for allowing effective time management, preventing possible laboratory errors, and providing more consistent test results (*3*, *4*).

Autoverification is a postanalytical process by which laboratory test results are released without manual intervention or review. Autoverification, which is an application of artificial intelligence for clinical laboratories, is thought to be an alternative to manual validation of test results (*4*). The first algorithm describing the use of computers to assist laboratory test validation was published over 50 years ago by Lindberg on the identification of “critical” results (*5*). This research is aimed at the evaluation of correlated analyses (*e.g.,* urea and creatinine (CREA)) with consistency checks and delta checks when test results exceed the defined limits. Currently, AV ensures a well-designed set of rules, and more specific algorithms have been developed (*2*, *6*, *7*).

Approved guidelines include important recommendations for laboratories needing guidance on the implementation of AV algorithms. The Autoverification of Clinical Laboratory Test Results: Approved Guidelines (AUTO 10-A and AUTO-15) were issued by the Clinical Laboratory Standards Institute (CLSI) in 2006 and 2019, respectively (*3*, *4*). These guidelines provide a basic template to allow each clinical core laboratory to build, implement, and validate specific AV rules for laboratory tests (*6*). However, it is recommended that each laboratory create the cut-off values required for autoverifying test results according to the needs of its patient population (*3*). Autoverification algorithms use various criteria to determine the reportability of test results. These criteria include instrument flags, QC checks, moving averages, serum indices, critical values, delta checks, and analytical measurement ranges (AMRs). Those algorithms can also examine patient or sample information from electronic medical records, times of sampling, and demographic information including age, sex, diagnosis, and inpatient/outpatient status (*8*).

Although the AUTO 10-A guidelines have been around for nearly 15 years, there remains a lack of standardisation, especially regarding algorithms, validation rules, and verification limits. In recent years, there has been an increasing body of literature on AV, but still, there is a limited number of studies on this topic in Turkey. In this respect, this study aims to contribute to the understanding of the AV process, provide well-designed AV rules that can be used in clinical laboratories, rapid and accurate verification of test results without human intervention, and raise work efficiency. It is the first time that we developed multi-rule algorithms for the verification of biochemical tests in Hatay Mustafa Kemal University (HMKU), Central Laboratory. We presented a detailed description of how to design an AV system that can be used as a starting point and for applying detailed system validation process. Indeed, our study, which was performed at a large district hospital with around 1700 inpatients and 24,000 outpatients treated *per* month, provides a new approach to the recent literature with respect to designing algorithms to detect pre-analytical errors through consistency checks.

## Materials and methods

### Design of AV algorithm

The AV process was carried out on ADVIA 1800 autoanalysers (Siemens Diagnostics, New York, USA) through the LIOS (Laboratory Information Operating System, Arbe Software Trading Company Ltd.) platform which was the middleware system. The middleware was used to provide the connection between the biochemistry autoanalysers and LIS. This electronic connection can process the preanalytical, analytical, and postanalytical data sent from the autoanalysers within the scope of the designed AV rules and send them to LIS. Additionally, the definition of AV algorithms and the creation of simulator patient data were completed through the middleware.

**Figure 1:**
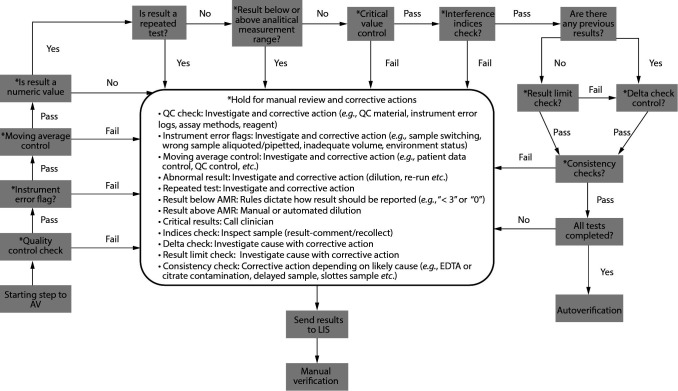
Master algorithm template for biochemical tests. QC – quality control. AMR – analytical measurement range. EDTA – ethylenediaminetetraacetic acid. LIS – laboratory information system. AV – autoverification.

While designing the AV algorithms, the evaluation criteria recommended in the approved guidelines were defined, and the master algorithm template was created (Figure 1). The corrective actions that were cause-specific for the laboratory tests that did not pass any algorithm rule are also given in Figure 1.

### Validation of AV

It should be validated that the developed algorithm follows the expected logic and produces the expected results. The validation of the AV system was performed in two phases. In the first phase, all algorithm criteria were applied to the 720 simulated results to follow the logic of the algorithm and verify the performance of the algorithm. The simulated results were created to include all algorithm decision rules for each biochemical test. In the second phase, the validation of the algorithm was performed using actual test results according to the AUTO-10A guideline (*3*). In this phase, 194,520 patient reports and 2,025,948 test results, which were previously assayed in our laboratory were collected from July 2019 to May 2020. We confirmed that the algorithms followed the expected logic, and data were recorded on the middleware after checking the correctness of the calculations.

### Criteria for algorithm

Internal QC and calibration: Internal QC is routinely performed in our laboratory and transferred from the autoanalyser to the middleware according to the Westgard QC multi-rules (*9*). If there are no QC results within 24 hours or if any QC rule is violated, the middleware stops the AV for all samples. Calibration periods and the last calibration dates of all tests were defined in the middleware calibration screen.

Instrument error codes (Flags): If any instrument flag is sent from the autoanalyser, the results are held for later manual verification.

Moving average: We used the ‘moving average’ method as an additional QC method to help provide the quality of the results. The moving averages involving the collection of over the last 20 patient results are analysed. Using these results, a mean value is defined, a warning limit which is mean ± 2 standard deviation (SD) is calculated, and then, a warning message is sent to the user *via* SMS (Short Message Service) (*6*, *10*).

Analytical measurement range (AMR): We used the AMRs recommended by the manufacturer for the autoanalyser (Siemens Diagnostics, New York, USA). If any results were outside the range of the AMR, a warning message was generated, and sample dilution was performed automatically or manually.

Critical Values: We used the critical values recommended by the General Directorate of Health Services, Department of Investigation, and Diagnostic Services (Ankara, Turkey) (*11*). If any results exceeded the range of the critical value, they were manually verified by a laboratory technician, and a phone call was immediately made to clinicians.

Serum Indices: The haemolysis, icterus and lipaemia (HIL) interferences were detected using multiple spectrophotometric readings by the biochemistry analyser in the serum specimen for the specific tests. If analytes like potassium (K), aspartate aminotransaminase (AST), and lactate dehydrogenase (LDH) were higher in the haemolytic samples that required examination by a technician, these results were held for manual verification.

Delta check: Delta check is performed to compare the present result for a patient to previous results and evaluate the probability of significant change. Delta check has been performed in previous studies using various methods such as delta difference, delta percent change, rate difference, and rate percent difference (*12*).

In this study, we chose the rate percent difference, which was calculated as the present value subtracted from the previous value then divided by the previous value, followed by dividing the results by the time interval. We chose a time interval as three months based on discussions with physicians at the Department of Medical Biochemistry. We used the reference change value (RCV), expressed as a percentage or absolute value which is a common systematic approach in the determination of delta thresholds (*9*).



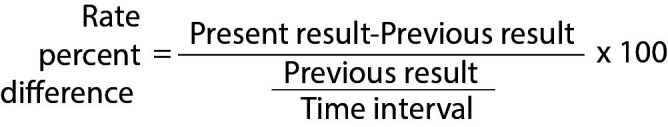



Result limit checks: Result limit checks are used to specify if the result exceeds predetermined thresholds. Three different limits were used to determine the thresholds as reference range, reference range ± total allowable error (TEa), and 2nd and 98th percentile values (*8*, *13*). Reference ranges were provided by the manufacturer, and we used them for each test according to the relevant reagent procedures. Total allowable error values that were taken from the College of American Pathologists (CAP) database were used for each test to determine reference range ± TEa values (*14*). For each test from the historical data of 2019, 2nd and 98th percentile values were calculated, and outliers were excluded by using box-blot analyses.

Consistency checks: Consistency rule checks are cross-checks established based on two or more different correlated tests. The cross-checks included in the algorithm in the last control step are given in Table 1.

**Table 1:** Consistency rule checks

Citrate contamination	Calcium decreased by 50% Sodium increased by 5 mmol/L Chloride decreased by 10 mmol/L
Clotted sample	Sodium < 136 mmol/L Potassium < 3.5 mmol/L Calcium < 8.4 mmol/L Glucose < 3.9 mmol/L
Ethylenediaminetetraacetic acid (EDTA) contamination	Potassium > 7 mmol/L and calcium < 8 mmol/L, or ALP < 50 U/L, or magnesium < 2 mmol/L
Delayed sample	Glucose < 2.2 mmol/L Potassium > 6 mmol/L Haemolysis < 50 mg/dL
Discordant results	ALT/AST ratio < 0.25 or > 4 Albumin / Total Protein ratio < 0.25 or > 1 Direct Bilirubin / Total Bilirubin ratio > 1 HDL cholesterol / Total Cholesterol ratio > 0.75
Intravenous glucose contamination	Sodium < 136 Chloride < 98 Potassium > 5 Glucose > 6.1
Intravenous saline contamination	Increased chloride normal sodium, low or critical low potassium
Monoclonal protein interference	High lipemia index (> +2) with low/normal triglyceride
Glomerular filtration rate	It is evaluated together with the creatinine test
Indirect bilirubin	It is evaluated together with direct bilirubin and total bilirubin
ALP – alkaline phosphatase. ALT – alanine aminotransaminase. AST – aspartate aminotransaminase. HDL cholesterol – high density lipoprotein cholesterol.	

### Comparison of manual review to AV

Patient reports, which were randomly selected by the middleware to compare manual review and AV results, were manually verified by seven expert reviewers. The number of the autoverified reports was 255 out of a total of 328 test reports, and 73 test reports were manually verified. The seven experts who performed the manual verification of the patient reports consisted of two medical biochemists, two medical biochemistry research assistant doctors, and three laboratory technicians with a wide range of professional knowledge. Using the obtained data, the AV and manual review results were compared statistically, and their agreement rate was evaluated. The AV system included 30 biochemical tests analysed in the Central Laboratory and most frequently ordered by clinicians (Table 2).

**Table 2:** Autoverification passing rates for all biochemical tests

**Biochemical parameters**	**Units**	**Reference range**		**Reference range ± %TEa**		**2nd and 98th percentile**		**Number of tests**
		Autoverify %	Stop AV %	Autoverify %	Stop AV %	Autoverify %	Stop AV %	
Alb	g/L	83	17	89	11	93	7	90,541
ALP	U/L	81	19	87	13	89	11	63,217
ALT	U/L	88	12	92	9	92	8	166,761
AMY	U/L	85	15	90	10	92	8	40,663
AST	U/L	83	17	87	13	91	10	142,545
BUN	mmol/L	81	19	84	16	87	14	148,408
CK	U/L	85	15	88	12	89	12	15,722
CK-MB	U/L	59	41	69	31	89	11	5379
DBIL	µmol/L	87	13	90	10	90	10	62,760
Fe	µmol/L	52	48	54	46	90	11	19,902
Phos	mmol/L	84	16	88	12	89	12	43,134
GGT	U/L	74	26	80	20	87	13	58,915
Glc	mmol/L	66	34	74	26	85	15	147,452
HDL	mmol/L	56	44	85	15	88	12	11,852
Ca	mmol/L	82	18	90	10	90	10	98,106
Cl	mmol/L	79	21	87	13	87	13	60,847
CHOL	mmol/L	68	32	80	20	91	9	11,672
CREA	µmol/L	65	35	79	21	79	21	173,905
LDH	U/L	71	29	80	20	91	7	57,363
LDL	mmol/L	42	57	63	37	93	7	12,691
Lip	U/L	84	16	88	12	90	10	37,945
Mg	mmol/L	87	13	87	13	87	13	47,594
K	mmol/L	64	36	68	32	68	32	140,852
Na	mmol/L	83	17	89	11	90	11	144,863
TBIL	µmol/L	84	16	85	15	89	12	63,069
TP	g/L	70	30	75	25	77	23	54,860
TRSF	g/L	23	77	37	63	89	12	130
TG	mmol/L	65	35	77	23	90	10	21,004
UIBC	µmol/L	55	45	78	22	90	10	19,544
UA	mmol/L	81	19	87	13	90	10	64,252
Total								2,025,948
Gray – indicate the highest passing rates for biochemical tests based on different result limit checks. TEa – total allowable error. AV – autoverification. Alb – albumin. ALP – alkaline phosphatase. ALT – alanine aminotransaminase. AMY – amylase. AST – aspartate aminotransaminase. BUN – blood Urea Nitrogen. CK – creatine kinase. CK-MB – creatine, direct kinase isoenzyme MB. DBIL – direct bilirubin. Fe – iron. Phos – inorganic phosphate. GGT – gamma-glutamyltransferase. Glc – glucose. HDL – high density lipoprotein cholesterol. Ca – calcium. Cl – chloride. CHOL – cholesterol. CREA – creatinine. LDH – lactate dehydrogenase. LDL – low density lipoprotein cholesterol. Lip – lipase. Mg – magnesium. K – potassium. Na – sodium. TBIL – total bilirubin. TP – total Protein. TRSF – transferrine. TG – triglyceride. UIBC – unsaturated Iron-Binding Capacity. UA – uric acid.								

The study was carried out with the approval of the HMKU Ethics Committee, with protocol numbered 2019/91, and resolution numbered 03. This study was supported financially by the HMKU Scientific Research Projects (BAP; project number: 19.U.016) Unit, for which we are thankful.

### Statistical analysis

All data of the study were analysed using the IBM SPSS Statistics software, Version 21.0, (SPSS Inc., New York, USA). P *<* 0.05 was considered significant. The categorical variables are expressed as counts and percentages. Second and 98th percentile values were calculated for each test from the historical data of 2019, and outlier values were eliminated by using box-blot analyses. Our results were statistically analysed according to three different result limit checks which were reference range, reference range ± TEa, and 2nd and 98th percentile values. Cohen’s κ value was calculated to determine the degree of agreement between seven independent reviewers’ assessments and the AV system. The κ coefficient ranges from - 1 to + 1. A κ value of + 1 indicates the highest agreement, while a κ value of - 1 indicates the highest disagreement between the users (*15*).

## Results

**Figure 2:**
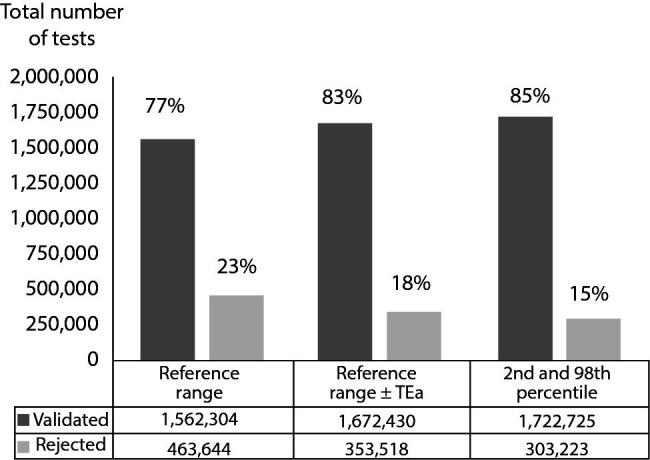
Autoverification passing rate on test-based according to different verify limits. Validated – It refers to the percentage of tests that were passed according to the algorithm criteria in Figure 1. Rejected – It refers to the percentage of tests that were not passed at least one of the algorithm criteria in Figure 1. TEa – total allowable error.

The test-based AV rate for all biochemical tests was 85% over a period of one year when the 2nd and 98th percentile values were used as result limit checks. When the reference ranges were used, the AV pass rate was found to be the lowest at 77% (Figure 2).

The test-based AV pass rate according to three different limit ranges are detailed in Table 2. The highest rates of AV were found for the alanine aminotransaminase (ALT), direct bilirubin (DBIL), and magnesium (Mg) parameters, which all had AV rates exceeding 85%. The least validated tests were transferrin (TRSF), low-density lipoprotein cholesterol (LDL), and iron (Fe) according to the reference range, and the AV pass rates of these parameters were 23%, 42%, and 52%, respectively. Using the 2nd and 98th percentiles as a limit range increased the AV pass rates for TRSF, LDL, and Fe by 65%, 51%, and 37%, respectively, and these were also the tests that showed the highest increase in their AV pass rates.

**Figure 3:**
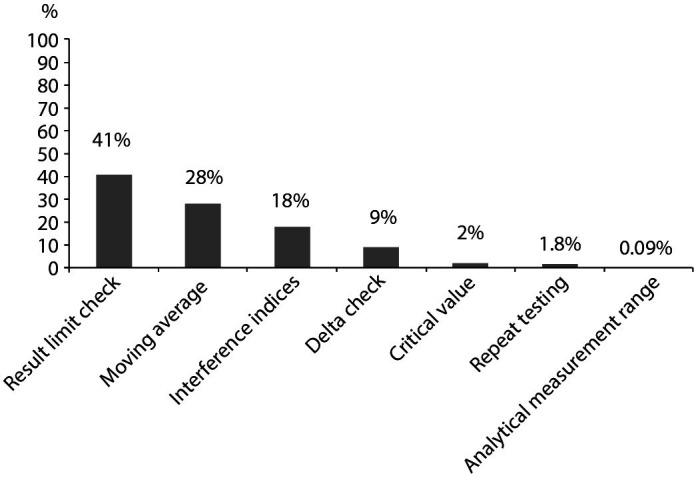
Cause analysis of manually verified test results. The reference range ± TEa was used as the decision limit. TEa – total allowable error.

In the AV process, the most common reasons for manually verified test results are given in Figure 3. A total of 2,025,948 tests were included, of which 83% (1,672,430) were autoverified when the reference range ± TEa criterion was used as the limit range (Figure 2). Result limit check was observed to be the most common reason for stopping AV (41%) (Figure 3). Result limit checks, which were the most common reason for non-validated results, were the same for all three different decision limits, and the most common reasons were result limit checks, moving averages, and serum indices, respectively.

A total of 328 actual patient reports were included, of which 255 (78%) were autoverified, and 73 (22%) were manually verified, according to the reference range ± TEa criterion. The degree of agreement between the users and the middleware was analysed using the κ statistic, and the results are given in Table 3. Accordingly, the statistical analysis resulted in a κ value between 0.39 and 0.63 (from minimal to moderate agreement, P < 0.001). The strongest agreement between user 1 and the middleware was found to be a statistically significant and moderate agreement (κ = 0.63; P < 0.001).

**Table 3:** Degree of agreement between the LIOS and each expert reviewer

**Reviewers**		**Autoverification Rules (LIOS)**		**Agreement**	**Sensitivity**	**Specificity**	**κ value**	**Kappa approximate significance**
		V	R					
Reviewer 1	V	243*	27^§^	88	90	79	0.63	P < 0.001
	R	12^‡^	46^†^					
Reviewer 2	V	249*	42^§^	85	86	84	0.49	P < 0.001
	R	6^‡^	31^†^					
Reviewer 3	V	234*	26^§^	86	90	69	0.58	P < 0.001
	R	21^‡^	47^†^					
Reviewer 4	V	234*	31^§^	84	88	67	0.52	P < 0.001
	R	21^‡^	42^†^					
Reviewer 5	V	210*	23^§^	79	90	53	0.46	P < 0.001
	R	45^‡^	50^†^					
Reviewer 6	V	240*	43^§^	82	85	67	0.41	P < 0.001
	R	15^‡^	30^†^					
Reviewer 7	V	247*	48^§^	83	84	76	0.39	P < 0.001
	R	8^‡^	25^†^					
*True positive. ^†^True negative. ^‡^False positive. ^§^False negative. Agreement – True Positive + (True Negative / Total number) x 100. Sensitivity – True Positive / (True Positive + False Negative) x 100. Specificity – True Negative / (True Negative + False Positive) x 100. κ value – 0-0.20 none; 0.21-0.39 minimal; 0.40-0.59 weak; 0.60-0.79 moderate; 0.80-0.90 strong; > 0.90 almost perfect (16). P < 0.001 – Highly statistically significant degree of agreement. V – validated. R – rejected. LIOS – laboratory information operating system.								

## Discussion

Autoverification is a powerful tool that uses rule-based systems to evaluate and validate test results without manual intervention. Currently, laboratories use AV in different groups of tests, including routine tests which are biochemistry, immunoassays, haematology, coagulation, blood gas, and urinalysis (*16*). It has apparent benefits in improving test quality, reducing error rates, decreasing turnaround time, and enhancing the efficiency of laboratory verification. In contrast, manual verification is a time-consuming activity with inherent subjectivity, and thereby, it cannot provide sufficiently accurate verification of test results (*17*). To overcome these limitations, we designed and implemented a middleware-based multi-rule system for AV in biochemical tests (Figure 1).

It is necessary to design AV rules for laboratories like our facility where approximately 1100 samples are analysed for routine biochemical tests. Biochemical tests constitute a large part of the analysed patient samples. When a large number of results in the queue are examined, fatigue can develop, and this is admittedly a potential risk factor for laboratory errors (*13*). In this regard, in our study, we developed algorithms to detect and minimise common pre-analytical errors including ethylenediaminetetraacetic acid (EDTA) or citrate contamination, clotted sample, and delayed sample through consistency rule checks (Table 1). In previous research, it has been reported that the most commonly used criteria in AV algorithms are AMRs, critical values, instrument error codes, serum indices, and delta check values (*18*, *19*). In our study, we also included moving averages, reference ranges, result limit checks, and consistency checks in the AV rules to create a more specific multi-rule algorithm (Figure 1). Therefore, the findings of this study suggested that test results were reviewed in more detail with a multi-rule algorithm.

The AV system in this study was designed according to three different decision limits that have been recommended in the literature (*6*, *13*, *20*). The AV passing rate was found between 77% and 85% based on the algorithms we developed. The highest AV passing rate was in the parameters of ALT, DBIL, and Mg, which all had rates exceeding 85%. Torke *et al.* used the midpoint between the median of the reference range and AMR as a limit range, and the AV passing rate they found increased to 62% (*19*). Our passing rates were higher than their system, at a verification rate of approximately 77%. A higher percentage of test results (83%) was autoverified based on the reference range ± TEa criterion. Previous studies have shown that by using the reference range ± TEa criterion, the AV passing rate varies between 73% and 85% (*8*, *20*). A study conducted by Shih *et al.* concluded that the rate of AV was 92% for 25,526 patient reports and 42 biochemical tests using the 2nd and 98th percentiles of cumulative patient data (*21*). We found that the AV rate obtained using historical patient data was lower (85%). These differences could be explained by differences in the limit ranges, the groups of tests that are studied, and the laboratory equipment that is used. Additionally, critical values and AMRs were not autoverified but held for later manual verification in our study. The least validated tests were TRFS, LDL, and Fe when the reference range criterion was used. One reason for this could be that the Fe test shows higher diurnal variation than the other tests (*22*). The reason for the variability in the LDL test was thought to be the differences in genetics and dietary habits in the population in which the study was conducted. The dramatic increase in the passing rate of the transferrin test was probably related to the lower number of test orders compared to other tests.

Several studies have recommended the use of delta checks in AV algorithms (*12*, *23*, *24*). In this study, the delta check limits were determined by using the RCV values obtained from the biological variation database (*2*). In our study, the delta check limit used for the CREA test was ± 13%, and the AV rates were between 65% and 79% (Table 2). A recent study by Gruenberg *et al.* revealed a higher AV passing rate, greater than 90%, using a ± 60% delta check for 23,410 CREA results (*25*). In a previous study in which delta check limits were evaluated as < 20%, AV rates between 50% and 75% were obtained. Understandably, the AV passing rates reported in multiple studies have shown differences (*7*, *26*, *27*). These differences were probably due to using different result limit checks and delta check limits and developments in AV rules over time.

In the AV process, one of the important issues is the cause analysis of manually verified test results. In our study, the most common reasons for non-validated results were result limit checks, moving averages, and serum indices, respectively. Rimac *et al.* reported that among 31 different biochemical tests, the least common reason for non-validated results was the critical value (2%) (*27*). Similarly, the same rate of critical value was 2% in our study, and this result was consistent with data reported in the literature.

The degree of agreement between AV and the seven expert reviewers’ assessments was found significant (agreement rates between 79% and 88%, P < 0.001), which pointed out that our system was valid (Table 3). Mohy-Sediq *et al.* compared AV system results to results provided by 4 reviewers, and the agreement rates were between 73% and 77%, which were lower than those in our study (*28*). The validation of AV rules is crucial in ensuring that the AV system operates as intended and requires high attention to detail. Additionally, there is a need for developing middleware that allows well-designed algorithms and validation (*29*).

This study had some limitations. As the comparison of AV and user results was evaluated on previously analysed and validated patient results, the discordant results could not be reanalysed. Prospective studies are needed for such an analysis.

Our experience suggests that designing and using a comprehensive AV algorithm requires carefully created rules and the performance of a well-designed validation process. The AV system based on a middleware enabled more rapid and routine evaluation of test results, minimised the requirements for manual work and provided more consistent test results. Since starting to use the AV system, our laboratory accelerated verification so we can save more time and focus on verifying the abnormal test results. Our improved model can help design, build, and validate an AV system and be used as a starting point for different test groups.
